# The effect of acrylamide on mitochondrial membrane potential and glutathione extraction in human spermatozoa: A laboratory study

**DOI:** 10.18502/ijrm.v13i10.7770

**Published:** 2020-10-13

**Authors:** Zeinab Omidi, Zeinab Piravar, Mina Ramezani

**Affiliations:** Department of Biology, Faculty of Sciences, Central Tehran Branch, Islamic Azad University, Tehran, Iran.

**Keywords:** Acrylamides, Spermatozoa, Mitochondrial membrane potential, Glutathione S-Transferase.

## Abstract

**Background:**

Acrylamide (AA) is a compound used in the industrial production of polyacrylamide. AAs affects by creating oxidative stress. It produces reactive oxygen species and leads to lipid peroxide. Lipid peroxidation in the cell membrane is one of the most important oxidations in the sperm, which can disrupt the fluidity and permeability of cell membranes and damage all cells.

**Objective:**

To investigate the different concentrations of AA on human sperm parameters based on the World Health Organization standard and its impact on mitochondrial membrane potential and sperm glutathione levels.

**Materials and Methods:**

In this laboratory study, we examined the different concentrations of AA on human sperm parameters based on the World Health Organization standard and its impact on mitochondrial membrane potential by flow cytometry and sperm glutathione levels by ELISA assay.

**Results:**

The results were reported as the mean fluorescence intensity of JC and the index was observed to decrease following the effect of AA in mitochondrial membrane potential (Δ Ψm). The results of ELISA test to study the level of intracellular glutathione showed that with the increase in the concentration of AA exposed to sperms, there was a significant reduction in the level of intracellular glutathione.

**Conclusion:**

AA destroys the sperm membrane integrity under apoptotic and oxidative inductions with a negative impact on mitochondrial function and antioxidative enzyme in sperm such as glutathione.

## 1. Introduction

Acrylamide (AA) with the formula C3H5NO is one of the compounds that are widely used in everyday life. This chemical is used for the industrial production of polyacrylamide. Polyacrylamide is a cleaning chemical used as flocculants in water and wastewater treatment. It is also used in the preparation of adhesives, papers, plastics, and cosmetics (1). Besides, AA are found in cigarette smoke and foods that are heavily heated, such as crispy bottom-of-the-pot foods, chips, and fried foods. Several previous studies have reported tumorigenesis of the substance in the thyroid gland, pancreas, kidneys, colon, uterus, and breast in mice and humans, as well as the development of disorders in the nervous system by AA (2). AA affects by creating oxidative stress, produces reactive oxygen species (ROS) and leads to lipid peroxide (3). Lipid peroxidation in the cell membrane is one of the most important oxidations in the sperm, which can disrupt the fluidity and permeability of cell membranes and damage all cells. Hence, lipid peroxidation not only directly causes the damage to membrane and its function but also indirectly affects the DNA and its health (4). One of the reasons for the poor performance of sperm and, as a result, infertility in men is oxidative stress. Human sperm is very vulnerable to these types of agents, because the sperm membrane is rich in unsaturated fatty acids that are susceptible to free radicals attack (5). Moreover, spermatozoa are very unique cells, because they lose most of their cytoplasm before releasing the germinal epithelium in the spermiogenesis stage. As a result, the cells have a small amount of cytoplasmic antioxidant enzymes such as glutathione peroxidase, catalase, and superoxide dismutase. While these enzymes are abundant in somatic cells, they help defend against oxidative damage (6).

Part of ROS is produced by the sperm itself and in low concentrations is essential for sperm activity, its capacitation, acrosome reaction, sperm-egg fusion, and other molecular events involved in the process of fertilization (7). The ROS produced by the sperm are superoxide anions, the high levels of which result in low sperm motility and infertility. In addition, it is also associated with damage to DNA, proteins, lipids, and carbohydrates. Lipid peroxidation of unsaturated fatty acids in the head and middle piece of the sperm, due to oxidative stress, changes sperm morphology and decreases motility and finally ineffectiveness of sperm-oocyte fusion reaction (7).

Mitochondria in human sperm are essential for the production of ATP in carrying out cellular processes, including motility and fertilization ability. ATP production in mitochondria takes place by oxidative phosphorylation reaction, compared to cytosolic ATPs (8). The accuracy of mitochondrial function, especially the high quality of the mitochondrial membrane potential (Δ Ψm), increases the antioxidant capacity of the cells. It is obvious that mitochondria are sites for superoxide production and, of course, proteins in Δ Ψm against oxidative stress. For this reason, their inhibition by oxidative agents increases the intracellular ROS and damage to the cell membrane and thereby reduces sperm motility (9). One of the defense systems against the damage caused by oxidative stress in human semen and sperm is the glutathione-s-transferase (GST) enzyme (10). The GST enzyme comprises a family of phase II enzymes involved in the detoxification of xenobiotic compounds (10). GST sperm enzyme activity results in damage to the membrane, which is associated with loss of mobility, inhibition of acrosome reaction, and reducing its ability for fertilizing egg outside the womb (11).

Although several studies have been conducted regarding the effect of AA on the reproductive system, especially its role on sperm function and morphology, there is no study on the impacts of this substance on Δ Ψm and its role in infertility. The aim of this study was, therefore, to examine the different concentrations of AA on human sperm parameters based on the WHO standard and its impact on Δ Ψm and sperm glutathione levels.

## 2. Materials and Methods

### Semen collection

In this laboratory study, 30 semen samples from fertile men who have a currently or formerly pregnant partner with known TTP (time to pregnancy) up to and including 12 months were collected after four days of deprivation of sexual activity. After transferring samples to the laboratory, the sperms were centrifuged at 500 g for 5 min and was added to the 10% Fetal Bovine Serum culture medium and recentrifuged. The sperms were divided into four groups as control and three groups treated with three (0.5, 1, and 2 mM) concentrations of AA (Merck KGaA diluted in the DMEM/F12 medium). Additionally, 200 μl of a solution containing sperm were added to 200 μl of AA solution. The solutions were incubated at 37°C for 4 hr. Sperm parameters were analyzed based on The World Health Organization guidelines (12).

### Evaluation of sperm motility and viability

In the present study, Computer Assisted Sperm Analysis was used to examine the sperm motility; 5 μl of sperm sample were placed on a special sperm chamber with a depth of 10 µ. Field images were transferred to a computer by a camera and the sample analysis was done. This machine samples in 1 sec at 50 different times (sampling frequency 50 HZ) from the sperm site. To assess the sperm motility, general information of sperm motility features including straight line velocity, curvilinear velocity, and amplitude of lateral head displacement was investigated (13). To identify motile and immotile live sperm from dead sperm, vital stain can be used. In this study, trypan blue stain was used; 10 λ trypan blue diluted was mixed with 10 λ supernatant of sperm and 2 λ formalin 10% diluted. The resulting solution was placed on a Neobar slide (Hemocytometer). Next, the sperms were studied through light microscopy. The number of live and dead spermatozoa was evaluated on five fields. The sperm membrane is impermeable to staining, so the dead sperms were stained and the living sperms were stainless. Because of the high human sperm speed, formalin was used as a fixative.

### Flow cytometry for the evaluation of mitochondrial membrane potential (Δ Ψm)

To evaluate the mitochondrial membrane potential of the cells by flow cytometry, sperms were centrifuged at around of 500 g for 3 min to separate the plasma from the cell pellet. The cell pellet was immersed in PBS (pH = 7.4), therefore, a solution with a number of about 1×10${}^{6}$ sperms per ml was achieved. Lipophilic cationic fluorescent dye JC-1 was used to evaluate the mitochondrial membrane potential of sperms (14). JC-1 had a unique feature in labeling the high- and low-membrane potential (Δ Ψm) of mitochondria. While JC-1 in high Δ Ψm became multimeric accumulation and emitted orange dye (with a wavelength of 590 nm), in low Δ Ψm, JC-1 became monomer and emitted green dye (between 525 and 530 nm). After spermatozoa were exposed to different concentrations of AA (0.5 mM, 1 mM, and 2 mM) for 4 hr, 5 × 10${}^{6}$ sperms were diluted in PBS, so that a concentration of 1.5-2 ml × 10${}^{6}$/ml for staining with JC-10.5 µl was achieved (concentration of JC-1 was 3 mM in DMSO). The samples were incubated at 37°C in the dark for 60 min. They were then analyzed with a flow cytometry (BD FACSCa Libur, made in USA, 2009), equipped with a 15 mW argon laser. The fluorochrome was used for fluorescein isothiocyanate and propidium iodide (PI). Thereafter, 10,000 cases at a velocity of 200-300 cells/s were recorded for each sample. Fluorescent green and orange were measured in FL1 and FL2 channels.

### Extraction of glutathione by enzyme-linked immunosorbent assay (ELISA) method

This test was used to evaluate the effect of AA on glutation extraction. All samples and solutions were required to be at an ambient temperature. Kit Basics: Two wells for each sample were considered (one well as a control and the other as a sample). In each well, 10 µL of the sample, 250 µL of solution 1, and 10 µL of solution 2 were added (both are soluble, specific, and confidential solutions of Zelbio Company). Then, 10 µL of distilled water was added. Thereafter, 20 µL of distilled water was added to each well and mixed; followed by a 20 µL of the chromogenic solution that was put and mixed in each well. The absorbance of the samples at 420 nm at 0 and 2 min was measured and read by ELISA (ELx800, BIOTEX Company, USA).

### Ethical consideration

Semen samples from fertile men were collected after four days of sexual abstinence from the Taleghani hospital, Tehran. Written informed consent was obtained from all subjects The Ethics Committee of Islamic Azad University approved the study protocol (code: IR.IAU.TMU.REC.1398.091).

### Statistical analysis

Data were analyzed using the SPSS computer software (SPSS Inc. V16.00, 2006, USA). Comparison between groups were made by one-way ANOVA followed by the Tukey's Kramer test. A P-value < 0.05 was considered statically significant.

## 3. Results

### Effect of acrylamide on sperm parameters

**Figure 1:**
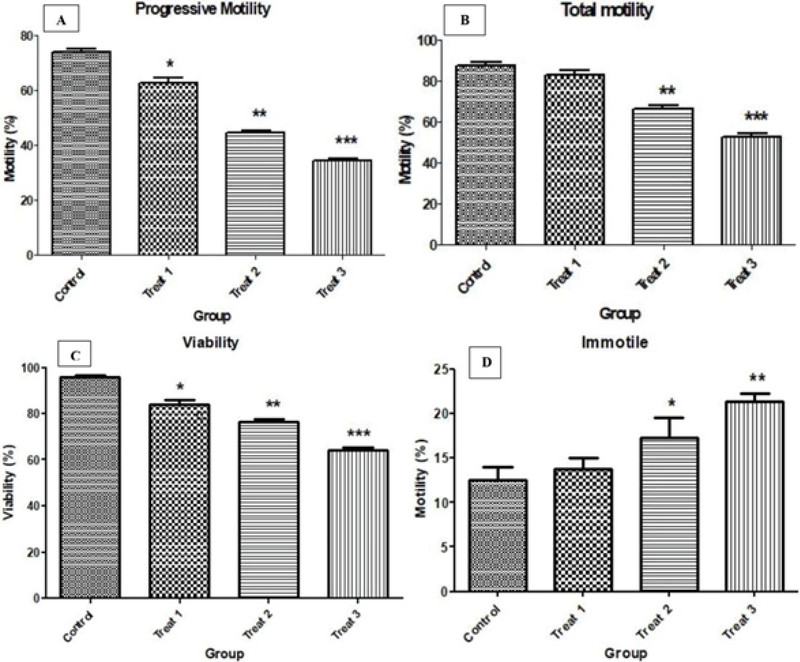
Comparison of progressive sperm motility percentage in groups treated with acrylamide compared to the control group (A). Comparison of total sperm motility percentage in groups treated with acrylamide compared to control group (B). Sperm viability percentage in groups treated with acrylamide compared to control group (C). Comparison of immotile sperm motility percentage in groups treated with acrylamide compared to control group (D). ${}^{*}$p < 0. 05; ${}^{**}$p < 0. 01; ${}^{***}$p < 0.001; CI > 95%.

The sperm analysis was performed according to the WHO international standard before and after treatment with AA. The effect of different concentrations of AA on total motility (progressive [PR] + nonprogressive motility [NP]) and PR motility of sperm showed a significant decrease in the trend of sperm motility compared to the control group (Table I). The progressive motility of sperms has shown a significant decrease with increase in the concentration of AA. While there was no significant difference in the total motility of the sperm between the concentration (0.5 mM) and the control group, the concentrations of 1 and 2 mµ AA showed a significant decrease in the total motility of the sperm as compared to the control group (Figure 1A, 1B). Viability and motility study of spermatozoa using trypan blue dye in all three concentrations of AA showed a significant decrease compared to the control group (Figures 1C, 1D).

### Mitochondrial membrane potential

**Figure 2:**
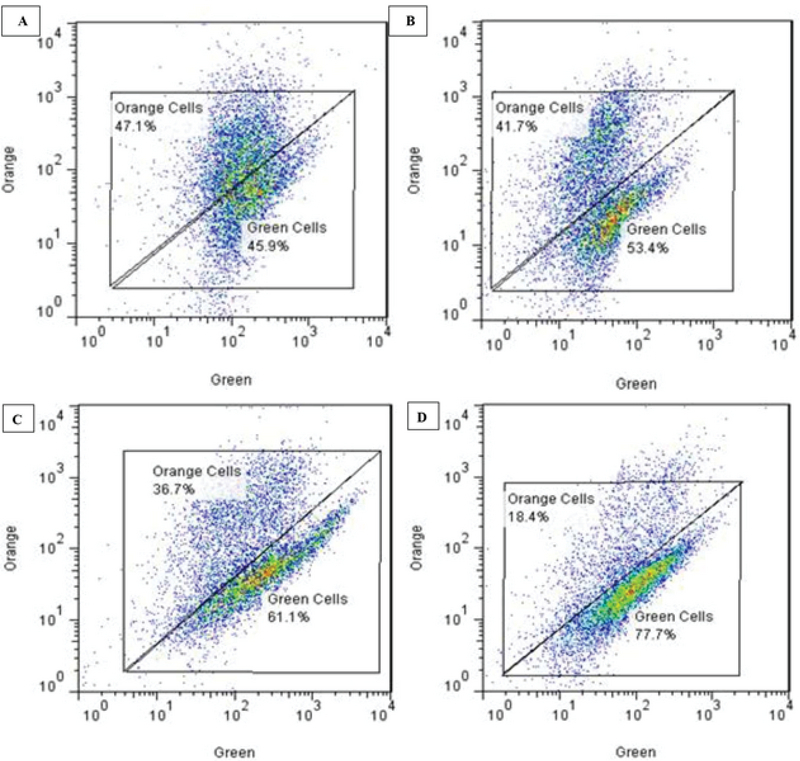
Comparison of the fluorescence intensity in (a) control group; (b, c, d) groups receiving acrylamide at a doses 0.5, 1, and 2 µM, respectively.

**Figure 3:**
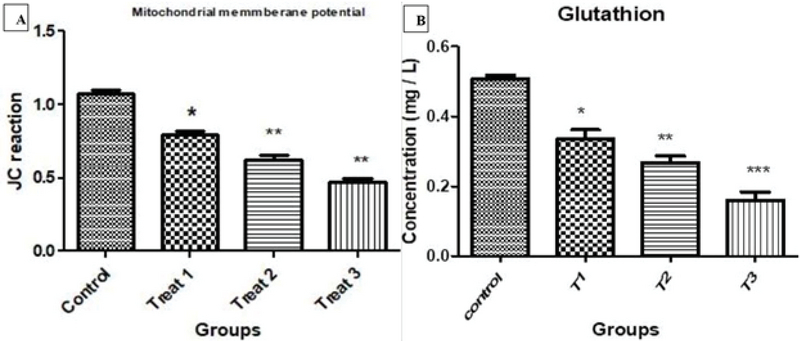
Comparison of the evaluation of sperm mitochondrial membrane potential (Δ Ψm) in groups treated with acrylamide compared to control group (A). Comparison of glutathione extraction in groups treated with acrylamide compared to control group (B). ${}^{*}$p < 0. 05; ${}^{**}$p < 0. 01; ${}^{***}$p < 0.001; CI > 95%).

Figure 2 shows the flow cytometric results of the effects of various concentrations of AA on the mitochondrial membrane potential Δ Ψm. The horizontal chart shows the green dye and the vertical chart shows the orange dye. The aggregation of cells near orange dye indicates normal Δ Ψm. With increase in the concentration of AA, cell aggregation tends toward the green dye, indicating a decrease in Δ Ψm. Figure 3A shows the progressive decrease of Δ Ψm with increasing concentration of AA.

### Extraction of glutathione

Because the GST enzyme is one of the antioxidant enzymes of the sperm and is considered as an indicator of sperm health, the results of ELISA test to study the level of intracellular glutathione show that with increase in the concentration of AA exposed to sperms, a significant reduction in the level of intracellular glutathione occurs, indicating a decrease in the intracellular GST activity (Table II, Figure 3B).

**Table 1:** The difference of mitochondrial membrane potential in acrylamide treatment and control groups by Tukey's Kramer test


**Tukey's multiple comparison test**	**Mean diff.**	**Significant**	**95% CI of diff.**
**Control vs. treat 1**	2.412	0.0001	0.9816-3.843
**Control vs. treat 2**	7.866	0.0001	6.435-9.296
**Control vs. treat 3**	13.49	0.0001	12.06-14.92
**Treat 1 vs. treat 2**	5.454	0.0001	4.023-6.884
**Treat 1 vs. treat 3**	11.08	0.0001	9.646-12.51
**Treat 2 vs. treat 3**	5.622	0.0001	4.192-7.053

**Table 2:** The difference of glutathione extraction parameter between human sperm in acrylamide treatment and control groups by Tukey's Kramer test


**Tukey's multiple comparison test**	**Mean diff.**	**P-value**
**Control vs. treat 1**	0.1719	0.002
**Control vs. treat 2**	0.2407	0.0001
**Control vs. treat 3**	0.3467	0.0001
**Treat 1 vs. treat 2**	0.06877	0.283
**Treat 1 vs. treat 3**	0.1748	0.008
**Treat 2 vs. treat 3**	0.1060	0.04

## 4. Discussion

Many studies have been conducted on the impact of AA on male fertility (15-17). Our information on the effect of AA on the mitochondrial membrane potential is very limited. In fact, our results include new information regarding the impact of AA on reduction of mitochondrial membrane potential and glutathione levels (18). Concerning the decrease in viability of AA-treated sperm, our results are consistent with the data of Kermani-Alghoraishi and colleague (1). Long-term exposure to AA can affect the development of spermatozoids and cause abnormal morphology and reduced viability (18). Moreover, AA, by affecting Leydig cells and secreting testosterone, influences their endocrine function and disrupts spermatogenesis (19). On the other hand, it has been shown that the amount of ROS production in viable cells is low. With regard to the above, it seems that decreasing the viability of cells by increasing the concentration of AA is due to an increase in intracellular superoxide anion (20).

Mitochondrial membrane potential is a valuable index to determine the health and function of cells. In a study performed on the relationship between mitochondrial membrane potential and sperm quality and fertility, it was shown that the high mitochondrial membrane potential indicates normal sperms with higher motility (7). Mitochondria are the source of intracellular ROS production. Coupling of electron transfer with oxidative phosphorylation can maintain the high membrane potential in mitochondria, which requires the production of ATP in cells (21). This process in sperm due to high production of ROS is damaged, and as a result, mitochondrial membrane potential is reduced. In the present study, mitochondrial membrane potential was evaluated through flow cytometry by JC. The lack of fluorescence is indicative of defects in the mitochondrial membrane potential. The results were reported as the mean fluorescence intensity of JC and it was observed that the index decreased following the effect of AA in spermatozoa. According to studies conducted in this case, it seems that decreasing the mitochondrial membrane potential due to increased ROS in the cell may be responsible for some of the sperm dysfunction and therefore infertility (22). AA destroys the sperm membrane integrity under apoptotic and oxidative inductions with a negative impact on mitochondrial function. Fat-soluble cations directly entering the mitochondrial matrix result in the production of superoxide anions and are primers of ROS production in mitochondria (23). With increasing concentrations of AA, the concentration of ROS increases. With this increase, Δ Ψm decreases simultaneously and the apoptosis pathway is activated by increasing the various enzymes of mitochondrial membrane such as caspase-9 and caspase-3 (24). Further, increase in ROS and decrease in Δ Ψm are associated with motility reduction, because electrophilic aldehyde lipids, in addition to the disruption of the electron current in the mitochondrial electron transport chain, can bind to the proteins that control the motility of the sperm (e.g., AKAP3, AKAP4, and dynein) and reduce the motility of the sperm following mitochondrial pro-oxidative and pro-apoptotic damage (23).

In addition, the increase in AA increases the peroxidation of the sperm plasma membrane lipids that reduces the fluidity of the membrane, resulting in decreased sperm motility. Some proteins in the outer membrane of mitochondria, like BH3, have pre-apoptotic activity and are inactive in normal state with low concentrations (25). If oxidative stimuli such as AA activate these proteins, expression of these proteins in the cell increases and leads to a series of reactions, ultimately resulting in the formation of pores in the mitochondrial outer membrane and its permeability. As a result, the electron leakage and its binding to compounds such as oxygen causes the production of superoxide anion (26). In the present study, following the incubation of sperms with AA, the reduction of Δ Ψm was observed. Since Δ Ψm is an important index regarding the optimal quality of sperms, their high motility and good fertility potential, decrease of Δ Ψm in relation to the influence of cytochrome C and the change of electron transport chain leads to disassembling of the proton gradient in the mitochondria inner membrane and reducing the ATP of the cell (27). Another explanation regarding the effect of AA on Δ Ψm is that AA inhibits the vital proteins on the mitochondrial inner and outer membrane. One of these proteins is the adenine nucleotide transporters on the inner mitochondrial membrane, which regulates the calcium channel, and inhibition of Δ Ψm causes the disruption of electron transport chain compounds and thus releasing of apoptotic agents from the membrane space into cytosol (28).

Oxidative and apoptotic damages also result in the loss of DNA integrity, so that different studies have reported the fragmentation of DNA in low concentrations of AA. Nuclear DNA fragmentation in the cell, directly or indirectly, results from the formation of pores in the mitochondrial membrane and the change in the mitochondrial membrane potential caused by an increase in intracellular ROS (29).

One of the important antioxidant enzymes in sperm is GST, and its inhibition results in damage to the cell membrane. As a result, it is associated with decreasing motility, inhibiting acrosome reaction, and reducing its ability to fertilize the oocyte (30). In this experiment, an increase in AA leads to depletion of glutathione reserves and a loss of balance between the oxidant and antioxidant system and provides conditions for the reduction of antioxidant reserves and the creation of lipid peroxidation process. That is why the reduction in glutathione stimulates the production of high levels of ROS that it would initiate intracellular signaling reactions in the mitogen-activated protein kinases family such as JUK. The kinases play a crucial role in regulating apoptosis and ultimately reducing cell numbers (31).

## 5. Conclusion

Due to the high consumption of compounds containing AA in everyday life, and increasing rate of infertility in human societies, results of this study indicate that different concentrations of AA has a decreasing and negative effect on human sperm parameters such as viability and motility, and that increasing the oxidative stress and production of ROS reduces the mitochondrial membrane potential and the levels of glutathione in the sperms. Thus, the reduction in the quality and quantity of sperm will result in the fertilization of oocyte. However, further studies are needed to prove the deleterious effects of AA on male fertility.

##  Conflict of Interest 

The authors declare that there is no conflict of interest in this study.

## References

[1] Kermani-Alghoraishi M, Anvari M, Talebi AR, Amini-Rad O, Ghahremani R, Miresmaeaili SM. The effects of acrylamide on sperm parameters and membrane integrity of epididymal spermatozoa in mice. *Eur J Obstet Gynecol Reprod Biol* 2010; 153: 52–55. 10.1016/j.ejogrb.2010.07.008 20705380

[2] Kumar J, Das S, Teoh SL. Dietary acrylamide and the risks of developing cancer: Facts to ponder. *Front Nutr* 2018; 5: 14–25. 10.3389/fnut.2018.00014 PMC5835509 29541638

[3] Azari A, Shokrzadeh M, Zamani E, Amani N, Shaki F. Cerium oxide nanoparticles protects against acrylamide induced toxicity in HepG2 cells through modulation of oxidative stress. *Drug Chem Toxicol* 2019; 42: 54–59. 10.1080/01480545.2018.1477793 29871546

[4] Najafi A, Amidi F, Sedighi Gilani MA, Moawad AR, Asadi E, Khanlarkhni N, et al. Effect of brain-derived neurotrophic factor on sperm function, oxidative stress and membrane integrity in human. *Andrologia* 2017; 49: 61–66. 10.1111/and.12601 27136309

[5] Santos Hamilton TRD, de Castro LS, Delgado Jde C, de Assis PM, Perez Siqueira AF, Mendes CM, et al. Induced lipid peroxidation in ram sperm: semen profile, DNA fragmentation and antioxidant status. *Reproduction* 2016; 151: 379–390. 10.1530/REP-15-0403 26811546

[6] Ferramosca A, Pinto Provenzano S, Montagna DD, Coppola L, Zara V. Oxidative stress negatively affects human sperm mitochondrial respiration. *Urology* 2013; 82: 78–83. 10.1016/j.urology.2013.03.058 23806394

[7] Aitken RJ, Muscio L, Whiting S, Connaughton HS, Fraser BA, Nixon B, et al. Analysis of the effects of polyphenols on human spermatozoa reveals unexpected impacts on mitochondrial membrane potential, oxidative stress and DNA integrity; implications for assisted reproductive technology. *Biochem Pharmacol* 2016; 121: 78–96. 10.1016/j.bcp.2016.09.015 27659810

[8] Ling X, Zhang G, Sun L, Wang Z, Zou P, Gao J, et al. Polycyclic aromatic hydrocarbons exposure decreased sperm mitochondrial DNA copy number: A cross-sectional study (MARHCS) in Chongqing, China. *Environ Pollut* 2017; 220: 680–687. 10.1016/j.envpol.2016.10.026 27751638

[9] Treulen F, Uribe P, Boguen R, Villegas JV. Mitochondrial outer membrane permeabilization increases reactive oxygen species production and decreases mean sperm velocity but are not associated with DNA fragmentation in human sperm. *Mol Hum Reprod* 2016; 22: 83–92. 10.1093/molehr/gav067 26621869

[10] Adeoye O, Olawumi J, Opeyemi A, Christiania O. Review on the role of glutathione on oxidative stress and infertility. *JBRA Assist Reprod* 2018; 22: 61–66. 10.5935/1518-0557.20180003 PMC5844662 29266896

[11] Kumar R, Singh VK, Atreja SK. Glutathione-S-transferase: role in buffalo (Bubalus bubalis) sperm capacitation and cryopreservation. *Theriogenology *2014; 81: 587–598. 10.1016/j.theriogenology.2013.11.012 24388131

[12] Cooper TG, Moonan E, Eckardstein SV, Auger J, Gordon Baker HW, Behre HM, et al. World Health Organization reference values for human semen characteristics. *Hum Reprod Update* 2010; 16: 231–245. 10.1093/humupd/dmp048 19934213

[13] Ariagno JL, Mendeluk GR, Furlan MJ, Sardi M, Chenlo P, Curi SM, et al. Computer-aided sperm analysis: a useful tool to evaluate patient's response to varicocelectomy. *Asian J Androl* 2017; 19: 449–452. 10.4103/1008-682X.173441 PMC5507091 27101803

[14] Sivandzade F, Bhalerao A, Cucullo L. Analysis of the mitochondrial membrane potential using the cationic JC-1 dye as a sensitive fluorescent probe. *Bio Protec* 2019; 9: e3128. 10.21769/BioProtoc.3128 PMC6343665 30687773

[15] Pourentezari M, Talebi A, Abbasi A, Khalili MA, Mangoli E, Anvari M. Effects of acrylamide on sperm parameters, chromatin quality, and the level of blood testosterone in mice. *Iran J Reprod Med* 2014; 12: 335–342. PMC4094659 25031578

[16] ALKarim S, ElAssouli S, Ali S, Ayuob N, ElAssouli Z. Effects of low dose acrylamide on the rat reproductive organs structure, fertility and gene integrity. *Asian Pacific Journal of Reproduction* 2015; 4: 179–187.

[17] Tyl RW, Marr MC, Myers CB, Ross WP, Friedman MA. Relationship between acrylamide reproductive and neurotoxicity in male rats. *Reproductive Toxicology* 2000; 14: 147–157. 10.1016/s0890-6238(00)00066-6 10825678

[18] Ma Y, Shi J, Zheng M, Liu J, Tian S, He X, et al. Toxicological effects of acrylamide on the reproductive system of weaning male rats. *Toxicol Ind Health* 2011; 27: 617–627. 10.1177/0748233710394235 21415092

[19] Yilmaz BO, Yildizbayrak N, Aydin Y, Erkan M. Evidence of acrylamide- and glycidamide-induced oxidative stress and apoptosis in Leydig and Sertoli cells. *Hum Exp Toxicol* 2017; 36: 1225–1235. 10.1177/0960327116686818 28067054

[20] Gosalvez J, Tvrda E, Agarwal A. Free radical and superoxide reactivity detection in semen quality assessment: past, present, and future. *J Assist Reprod Genet* 2017; 34: 697–707. 10.1007/s10815-017-0912-8 PMC5445049 28341974

[21] He B, Guo H, Gong Y, Zhao R. Lipopolysaccharide-induced mitochondrial dysfunction in boar sperm is mediated by activation of oxidative phosphorylation. *Theriogenology* 2017; 87: 1–8. 10.1016/j.theriogenology.2016.07.030 27587273

[22] Agnihotri SK, Agrawal AK, Hakim BA, Vishwakarma AL, Narender T, Sachan R, et al. Mitochondrial membrane potential (MMP) regulates sperm motility. *In Vitro Cell Dev Biol Anim* 2016; 52: 953–960. 10.1007/s11626-016-0061-x 27338736

[23] Chai RR, Chen GW, Shi HJ, Wai-Sum O, Martin-DeLeon PA, Chen H. Prohibitin involvement in the generation of mitochondrial superoxide at complex I in human sperm. *J Cell Mol Med* 2017; 21: 121–129. 10.1111/jcmm.12945 PMC5192824 27558591

[24] Juárez-Rojas AL, García-Lorenzana M, Aragón-Martínez A, Gómez-Quiroz LE, Retana-Márquez Mdel S. Intrinsic and extrinsic apoptotic pathways are involved in rat testis by cold water immersion-induced acute and chronic stress. *Syst Biol Reprod Med* 2015; 61: 211–221. 10.3109/19396368.2015.1030473 25867867

[25] Nichi M, Rijsselaere T, Losano J, Angrimani D, Kawai G, Goovaerts I, et al. Evaluation of epididymis storage temperature and cryopreservation conditions for improved mitochondrial membrane potential, membrane integrity, sperm motility and in vitro fertilization in bovine epididymal sperm. *Reprod Domest Anim* 2017; 52: 257–263. 10.1111/rda.12888 27925340

[26] Ahmad M, Ahmad N, Riaz A, Anzar M. Sperm survival kinetics in different types of bull semen: progressive motility, plasma membrane integrity, acrosomal status and reactive oxygen species generation. *Reprod Fertil Dev* 2015; 27: 784–793. 10.1071/RD13400 24576435

[27] Mughal IA, Irfan A, Hameed A, Jahan S. Sperm mitochondrial DNA 15bp deletion of cytochrome c oxidase subunit III is significantly associated with human male infertility in Pakistan. *J Pak Med Assoc* 2016; 66: 3–7. 26712170

[28] Barbonetti A, Castellini C, Di Giammarco N, Santilli G, Francavilla S, Francavilla F. In vitro exposure of human spermatozoa to bisphenol A induces pro-oxidative/apoptotic mitochondrial dysfunction. *Reprod Toxicol* 2016; 66: 61–67. 10.1016/j.reprotox.2016.09.014 27686954

[29] Malić Vončina S, Golob B, Ihan A, Kopitar AN, Kolbezen M, Zorn B. Sperm DNA fragmentation and mitochondrial membrane potential combined are better for predicting natural conception than standard sperm parameters. *Fertil Steril* 2016; 105: 637–644. 10.1016/j.fertnstert.2015.11.037 26696300

[30] Hering DM, Lecewicz M, Kordan W, Majewska A, Kaminski S. Missense mutation in glutathione-S-transferase M1 gene is associated with sperm motility and ATP content in frozen-thawed semen of Holstein-Friesian bulls. *Anim Reprod Sci* 2015; 159: 94–97. 10.1016/j.anireprosci.2015.06.001 26091956

[31] Silva JV, Freitas MJ, Correia BR, Korrodi-Gregório L, Patrício A, Pelech S, et al. Profiling signaling proteins in human spermatozoa: biomarker identification for sperm quality evaluation. *Fertil Steril* 2015; 104: 845– 856. 10.1016/j.fertnstert.2015.06.039 26209830

